# Supplementary material for: The role of medicines and therapeutics committees structure in supporting optimal antibacterial use in hospitals in Uganda: A mixed method study
Source: PLoS One. 2024 Jan 19;19(1):e0289851. doi: 10.1371/journal.pone.0289851 (PMC10798471; doi:10.1371/journal.pone.0289851)
Supplement: S1 File — (DOCX) [file pone.0289851.s001.docx]

# Additional file 3_Interview administered questionnaire on Structure and functional roles of Medicines and Therapeutic Committees (MTC) in optimizing antibacterial use in children under-five in selected Health facilities in Uganda

**To be administered:** *Chairperson , secretaries or clinicians who are members of the members of Medicines and Therapeutic Committee in all selected health facilities*

**Purpose**

To determine the structure and role of the Medicines and Therapeutic Committee in optimizing antibacterial use among children under-five in selected health facilities of Uganda

**Instruction**

- For a Yes or No question (e.g. is there a pharmacy at your health facility) please Mark using a tick in a box representing “**YES**” or “**NO”**.
- If the question asks for the “Number” (e.g. “How many weighing scales are there?”), please simply write the number of items in the appropriate box.
- If a question requires a comment, please give a comment representing your answer representing Yes or No

**Abbreviations**

- **MTC**: Medicines and Therapeutics Committee
- **CME**: Continuous Medical Education
- **STG**: Standard Treatment Guidelines
- **PNFP**: Private Not for Profit

**Questionnaire no. (pre-fill)…………………………………..**

| Date (pre-fill) | **………/………/20………………** |
| --- | --- |
| Data collector name (pre-fill intials of your name | **…………………………………..** |

**Please complete the information about health facility**

| 1. Health facility ID (*pre-fill*) | **…………………………….** |
| --- | --- |
| 1. City /town (*pre-fill)* | **……………………………** |
| 1. District (*pre-fill)* | ………………………….. |
| 1. Region *(pre-fill)* | ………………………………. |
| 1. Type of Health facility (*Tick one only)* | 1.  Regional referral hospital 2.  District hospital 3.  Private Not for profit hospital |
| 1. Nature of the health facility | 1.  Teaching hospital. . 2.  Non-teaching hospital . |
| 1. Number of licensed beds (pre-fill) ……………………… | 1. Number of admissions per-month (pre-fill)   ……………………………… |

1. **Respondent’s socio-demographics**

| 1. Respondent’s ID (pe-fill) | ………………………………… |
| --- | --- |
| 1. Sex | 1. Male 2. Female |
| 1. Age | ………………. (in years) |
| 1. Qualification /Position held | 1.  Nurse 2.  Medical doctor 3.  Specialist 4.  Consultant |
| 1. Marital status | 1. Single 2. Married 3. Divorced/widowed 4. Others…………………………………… |
| 1. Unit or department of work .. | 1. Medicine 2. Surgery 3. Paediatrics 4. Pharmacy 5.  Obstetrics and Gynaecology 6. Accident and Emergency 7. Out-patient Department 8.  Private department 9. Orthopaedic department 10. Other……………………….. |
| 1. Chairperson of the MTC | 1. Medical specialist  2. Pharmacist  3. Others …………….. |
| 1. Secretary of the MTC | 1. Medical specialist  2. Pharmacist  3. Others …………….. |
| 1. Years of practice. ……………………………… |  |

1. **Structure of Medicines and Therapeutic committees**

| 1. Does your hospital have Medicines and Therapeutic Committee (MTC) or equivalent? | 1. Yes  2. No  |
| --- | --- |
| 1. If yes, how many years has the MTC been established?................................................ |  |
| 1. How many subcommittee does you MTC have? | 1. Yes  2. No  |
| 1. How often does you MTC meet? | ………………………….. |
| 1. Which of the following categories of professionals’ form part of your committee? (please tick all that apply) | 1. Clinical Specialists representing the hospital departments (medical specialists) 2. Pharmacologist 3. Pharmacist 4. Nursing representatives 5. Microbiology 6. Finance and Administration |
| 1. Does the committee have an established place in the organizational structure of the hospital? | 1. Yes  2. No  |
| 1. Which of the following sub-committees does your MTC have? (please tick to select) | 1. supply chain management /Formulary committees 2. Antimicrobial committee / Antimicrobial stewardship committee 3. Infection control committee 4. Adverse drug reactions /pharmacovigilance committee 5. Other   ………………….. |
| 7. Are the roles and responsibilities of the chairperson, members of the committee, and the secretariat clear to you as member of this hospital? | 1. Yes  2. No  |
| 1. Are the goals and purpose of MTC clear to you as a member of staff of this hospital? | 1. Yes  2. No  |
| 1. Are MTC activities and interventions supported with by hospital administration or management? | 1. Yes  2. No  |

1. **Functional role of the MTC in optimizing antibacterial use in the hospital**

| 1. Has the MTC guided prescribers concerning the selection of antibacterials for the formulary in this hospital? | 1. Yes  2. No  |
| --- | --- |
| 1. Has the MTC developed policies/procedures on prescribing antibacterials in the formulary in this hospital? | 1. Yes  2. No  |
| 1. Has the MTC developed procedures for ordering antibacterials on the formulary? | 1. Yes  2. No  |
| 1. Has the MTC developed or adopted standard treatment guidelines (STG) to manage bacterial infection? | 1. Yes  2. No  |
| 1. Has the MTC assessed antibacterials use problems on your ward or in this hospital | 1. Yes  2. No  |
| 1. Have the MTCs advised or guided health workers on managing adverse drug reactions from antibacterials? | 1. Yes  2. No  |
| 1. Has the MTC advised on the prevention of medication errors from use or administration of antibacterials | 1. Yes  2. No  |
| 1. Has the MTC disseminated information on medicines use more especially antibacterials agents | 1. Yes  2. No  |

1. **Activities performed by the MTC to optimize use of antibacterial in your ward**

| - - - 1. **Formulary identification, selection, evaluation** | |
| --- | --- |
| 1. **Evaluating and selecting antibacterials for the formulary list** | 1.Yes  2. No  |
| 1. MTC has established a procedure for receiving requests for the addition or deletion of antibacterials on the formulary list | 1.Yes  2. No  |
| 1. MTC disseminates information to all members of staff on newly added or deleted medicines on the formulary | 1.Yes  2. No  |
| 1. MTC selects antibacterials based on defined criteria (essential medicine list, standard treatment guidelines, antibacterial resistance patterns) | 1.Yes  2. No  |
| 1. MTC has established a procedure of using antibacterials, not on the formulary | 1. Yes  2. No  |
| 1. MTC has established a procedure for managing drug product shortages, especially antibacterials | 1. Yes  2. No  |
| 1. MTC conducted regular audits on the stock inventory of movement of antibacterials and informed healthcare providers on the use | 1. Yes  2. No  |
| 1. **Improved safety of antibacterials through pharmacovigilance** | 1. Yes  2. No  |
| 1. MTC has identified, assessed, and reported on adverse drug reactions and medication errors from antibacterials | 1. Yes  2. No  |
| **Implemented antimicrobial stewardship activities to optimise antibacterial use** | 1. Yes  2. No  |
| 1. MTC educates healthcare professionals through CME and small group meetings on appropriate antibacterial use to improve clinical outcomes and safety. | 1. Yes  2. No  |
| 1. Updated and developed standard treatment guidelines and treatment algorithms to follow when managing infectious diseases | 1. Yes  2. No  |
| 1. MTC periodically evaluates the use of medicines in the hospital, adherence to standard treatment guidelines and provides feedback on medicine use problems in the hospital | 1. Yes  2. No  |
| 1. MTC conducts developed a policy for the restriction and approval of reserved antibacterial | 1. Yes  2. No  |
| 1. MTC developed structured antibacterials forms or preprinted order forms for use when ordering or dispensing certain antibacterials in specific conditions and on certain wards | 1. Yes  2. No  |
| 1. MTC has developed guidelines for dispensing antibacterial that restrict prescription by qualification | 1. Yes  2. No  |
| 1. **Development of drug policies, standard operating procedures** | 1. Yes  2. No  |
| 1. Implements a policy to ensure the availability of high-quality antibacterials in the hospitals | 1. Yes  2. No  |
| 1. Policy to assess, track, monitor, track and regulate expenditures on medicines like antibiotics | 1. Yes  2. No  |
| 1. Policy on drug promotion and guidance of Pharmaceutical representatives influence promotion | 1. Yes  2. No  |
| 1. Implements a policy to ensure the availability of high-quality antibacterials in the hospitals | 1. Yes  2. No  |
| 1. Policy to assess, track, monitor, track and regulate expenditures on medicines like antibiotics | 1. Yes  2. No  |
| 1. Policy on drug promotion and guidance of Pharmaceutical representatives influence promotion | 1. Yes  2. No  |

1. **MTC guidance on non-formulary use of antibacterials**

|  | Never | 2 | 3 | 4 | Almost always |
| --- | --- | --- | --- | --- | --- |
| 1. Does your MTC approve Therapeutic Interchanges for antibacterials that are not listed on the Formulary? |  |  |  |  |  |
| 1. How often are approved Therapeutic Interchanges performed by pharmacy staff when a non-formulary antibacterial is ordered? |  |  |  |  |  |
| 1. How often do pharmacy staff members call physicians to obtain an antibacterial order of a non-formulary medication instead of antibacterial on the formulary? |  |  |  |  |  |
| 1. How often do prescribers on ward contact members of the MTC to obtain a change of a formulary antibacterial to non-formulary medication? |  |  |  |  |  |
| 1. When a change is made to the formulary antibacterials, does the MTC publish printed communications on the changes (newsletters, memos, etc.)? |  |  |  |  |  |
| 1. When a change is made to the formulary how often does your hospital provide electronic communications (E-mails, on-line newsletters, etc) to prescribers? |  |  |  |  |  |
| 1. How often does a member of the MTC communicate formulary changes to individual or groups of prescribers? |  |  |  |  |  |
| 1. How often is information about formulary changes included as part of the order entry process? |  |  |  |  |  |

1. **MTC strategies to evaluate and prevent Adverse Drug Reactions (ADR) in antibacterials**

| 1. Has the MTC or its member guided prescribers on ‘high risk’ antibacterial agents that need to be monitored closely on the formulary lists | 1. Yes  2. No  |
| --- | --- |
| 1. Has the MTC or its member identified ‘high risk’ patient populations and monitored their treatment closely with antibacterials agents. (Such patients include pregnant women, children and patients with renal or liver dysfunction) | 1. Yes  2. No  |
| 1. Has the MTC educated prescribers about ADRs on antibacterials through in-service education, drug information bulletins and reports of collected adverse events. | 1. Yes  2. No  |
| 1. Does MTC have an established a group of clinicians including medical doctors, nurses and pharmacists to select best practices on antibacterial administration that prevent ADR | 1. Yes  2. No  |
| 1. Does the MTC have written procedures or guidelines or checklists for the administration of high risk intravenous antibacterials | 1. Yes  2. No  |
| 1. Has MTC reviewed ADR reports regularly and informed clinicians on incidence and impact of antibacterial ADRs in the region. | 1. Yes  2. No  |

1. **MTC interventions or strategies to optimize antibacterial use in bacterial infections**

| **Educational strategies** | |
| --- | --- |
| 1. Does the MTC conduct in-service education programs, workshops, seminars to guide you on rational antibacterial use? | 1. Yes  2. No  |
| 1. Has the MTC established key opinion leaders to mentor junior staff on prescribing antibacterials? | 1. Yes  2. No  |
| 1. Does the MTC organize CME and CPD on antibacterial use? | 1. Yes  2. No  |
| 1. Has the MTC established drug information resource center/unit with resources on antibacterial use? | 1. Yes  2. No  |
| 1. Has the MTC availed drug newsletters, manuals and bulletins to guide on antibacterial use? | 1. Yes  2. No  |
| 1. Does the MTC conduct training on use of developed standard treatment guidelines in bacterial infection? | 1. Yes  2. No  |
| **Managerial strategies** |  |
| 1. Has the MTC adopted or developed or implemented standard treatment guidelines on antibacterial use in children? | 1. Yes  2. No  |
| 1. Has the MTC conducted an audit (assessed) and given feedback on antibacterial use to prescribers? | 1. Yes  2. No  |
| 1. Does the MTC conducted programs which support pharmacy personnel on how guide clinicians on optimal use of antibacterials? | 1. Yes  2. No  |
| 1. Has MTC imposed a policy on restricting prescribing, dispensing of certain antibacterials among certain clinicians? | 1. Yes  2. No  |
| 1. Are there important strategies being implemented to improve antibacterial use? | 1. Yes 2. No  |
| 1. Does the MTC limit the availability certain antibacterials used in infections (e.g., broad spectrum antibiotics or expensive agents) in the facility? | 1. Yes 2. No  |
| 1. Does the MTC rotate antibacterial in and out of the formulary to contain antibacterial resistance? | 1. Yes 2. No  |
| 1. Does the MTC conduct retrospective review to evaluate how specific antibacterial have been used in infection? | 1. Yes 2. No  |
| 1. Has the MTC developed structured antibacterials forms or preprinted order forms for use when ordering or dispensing certain antibacterials in specific conditions, such as sepsis? | 1. Yes 2. No  |
| 1. Does the MTC have a policy for automatic stop orders (e.g., discontinuation of antibiotics after a specific time period)? | 1. Yes 2. No  |
| 1. Does the facility require approval from a supervising doctor, head prescriber, or pharmacist for the use of certain antibacterial? | 1. Yes 2. No  |
| 1. Does the MTC liaise with the infection control committee with regarding assessment and use of data from the monitoring of antimicrobial sensitivity and resistance patterns in hospitals and primary care clinics. | 1. Yes 2. No  |

**J. Influence of MTC members on optimizing antibacterial use on their wards**

|  | Not much influence | 2 | 3 | 4 | A great deal of influence |
| --- | --- | --- | --- | --- | --- |
| 1. How much influence do MTC members have on rational use of antibacterials on their wards? |  |  |  |  |  |
| 1. How much influence do MTC member have on improving patient outcomes on this ward? |  |  |  |  |  |
| 1. How much influence do MTC members have on reduction of antibacterial resistance in their wards? |  |  |  |  |  |
| 1. How much influence do MTC members have on communications and adoption of new antibacterial on the formulary? |  |  |  |  |  |
| 1. How much influence do MTC members have on setting the agenda for reviewing antibacterials for addition or deletion on the formulary? |  |  |  |  |  |
| 1. How much influence do MTC members have on the gathering information about antibacterial use on their wards for committee decisions? |  |  |  |  |  |

**K. Outcomes of MTCs interventions on optimizing of antibacterial use and reducing antibacterial resistance**

|  | Strongly  Agree | Agree | Somewhat  Agree | Disagree |
| --- | --- | --- | --- | --- |
| 1. MTC has increased selection of effective, safe, high-quality, and cost-effective antibacterials for the formulary |  |  |  |  |
| 1. MTCs has increased identification of antibacterial use problems and |  |  |  |  |
| 1. MTC has improved antibacterials use through a number of interventions |  |  |  |  |
| 1. MTC establishment has improved quality of patient care and health outcomes |  |  |  |  |
| 1. MTC reduced development of antibacterial resistance on wards through many controls |  |  |  |  |
| 1. MTC has increased staff and patient knowledge on antibacterial use and resistance |  |  |  |  |
| 1. MTC has decreased incidences of adverse drug reactions (ADRs) and medication errors with improved management |  |  |  |  |
| 1. MTC has improved medicine procurement and inventory management |  |  |  |  |
| 1. MTC has reduced pharmaceutical expenditures through better management of medicines |  |  |  |  |

**L. challenges faced by the MTC is facing optimizing antibacterial use in management of child hood infections?**

|  | Strongly  Agree | Agree | Somewhat  Agree | Disagree |
| --- | --- | --- | --- | --- |
| 1. Formulary list of antibacterials is not followed |  |  |  |  |
| 1. Prescribers do not know about antibacterial on the formulary list |  |  |  |  |
| 1. Prescribers do not believe in antibacterials on the formulary list |  |  |  |  |
| 1. There inconsistency between the antibacterials on the formulary list and those in the standard treatment guidelines (STGs) |  |  |  |  |
| 1. Prescribers don’t follow standard treatment guidelines (STGs) when prescribing antibacterials |  |  |  |  |
| 1. No STGs exist, or those that are available are they are outdated |  |  |  |  |
| 1. Prescribers do not know about the STGs |  |  |  |  |
| 1. Prescribers do not believe in following STGs when managing bacterial infections |  |  |  |  |
| 1. Inconsistency between the STGs and formulary list of medicines |  |  |  |  |
| 1. There are Frequent antibacterials stock-outs of due to high consumptions, unreliable suppliers, insufficient budget |  |  |  |  |
| 1. There is increased overuse and irrational use of antibacterials in the hospital’s medicines |  |  |  |  |
| 1. Prescribers like to use of overly expensive second line or third line antibacterials |  |  |  |  |
| 1. Lack of staff knowledge on rational antibacterial use |  |  |  |  |
| 1. Poor communication, for example, handwriting and oral orders; prescribers do not know how to write prescriptions properly |  |  |  |  |
|  |  |  |  |  |

Others specify

_______________________________________________________________________________________________________________________________________________________________________________________________________________________________________________________________
