# Supplementary material for: The role of medicines and therapeutics committees structure in supporting optimal antibacterial use in hospitals in Uganda: A mixed method study
Source: PLoS One. 2024 Jan 19;19(1):e0289851. doi: 10.1371/journal.pone.0289851 (PMC10798471; doi:10.1371/journal.pone.0289851)
Supplement: S2 File — (DOCX) [file pone.0289851.s002.docx]

# Additional file 1_Interview guide on the role of MTC in optimizing antibacterial use in children under-five in selected health facilities in Uganda

**Interviewer: ____________________ Hospital I.D____________________.**

**Date of Visit: __________________**

Greeting

***Statement of confidentiality***

1. (Welcome participants to the interview and thank them for their willingness to participate)

Thank you very much for agreeing to be interviewed. I am a PhD candidate at Makerere University and evaluating the structure, activities and drug selection process used by Medicines and Therapeutics Committees in selected Health facilities of Uganda.

2. (Explain the purpose of the study and obtain written Consent to participate in the study).

Would you mind if I record our conversation, as it would be difficult to capture everything you say? Everything you say will be confidential, and your name will not be mentioned anywhere. If you agree, would you kindly sign the consent form?

3. Encourage the participant to speak freely

*Interviewer Note: Start the audio recorder to record the interview*

4. Pose the questions to the participant

1. **Nature of the behaviors**
2. How easy or difficult has it been to manage MTC of this hospital?

***Prompts***

- *Who are its members?*
- *What are your thoughts necessary behaviours of members to conduct activities of the MTC?*

1. What are some of the functions of this MTC?

- ***Prompt:*** *Which members are responsible for those functions /what expertise is needed for some of those functions?*

1. **Knowledge**
2. How easy or difficult is it to access antibacterials on the formulary list by the clinician?

***Prompts:***

- *How has the MTC guided the clinician on the use of antibacterials on the formulary?*
- *How has the MTC guided clinicians on the non-formulary use of antibacterials on their wards*

1. What strategies has the MTC put in place to improve the use of antibacterials in this hospital?

***Prompts:*** *How easy or difficult is it to implement some of these strategies?*

*Probe these…below*

*(Empirical antibacterial treatment, use of combinational antibiotics, provided education to the clinician on how to use antibiotics, use of locally developed antibacterial guidelines, consulting local infectious disease expert or specialist)*

1. What strategies have the MTC used to limit unnecessary antibacterial use in this hospital?

***Prompts****: How easy or difficult is it to use some of these strategies?*

*Probe these…below*

*(de-escalation approach to therapy, avoid prolonged use of prophylactic antibacterial , apply selective formulary control or restriction of some antibacterials, develop and apply local guidelines)*

1. What outcomes has the MTC generated from implementing the above strategies?
2. What challenges are faced by the MTC in implementing these strategies in the hospital?
3. **Skills**
4. How much expertise or experience do you think one needs to participate as a member of MTC?
   - ***prompt****: tells us some of the expertise needed for MTC activities. Does one need some training, years of experience…... Tell us more*
5. Do you think any special skills or expertise are needed when MTC is selecting antibacterials for the formulary of hospitals?
6. What type of skills and experience do you think MTCs members have in implementing activities or strategies on improved use of antibacterials in hospitals?

**D. BELIEFS ABOUT CAPABILITIES**

- How confident are you about MTC solving inappropriate antibacterial use in this hospital?

**E. OPTIMISM**

- How optimistic are you that in the future MTC will solve this hospital's inappropriate antibacterial use medicines selection problems?

**F. BELIEFS ABOUT CONSEQUENCES**

- What do you think happen if the MTC did not antibacterials select medicines needed for their conditions well?
- What do you think might happen to you if YOU didn’t have essential medicines to manage the conditions of your patients?
- What do you think might happen to other members of your team if YOU didn’t take the right procedures as MTC to select the needed medicines of the hospitals?

**G. REINFORCEMENT**

- Is there anything that would encourage or discourage you as Member of Medicines and therapeutic committee when selecting medicines for the formulary list?

Prompts: incentives, sanctions, appraisal, care review

H. **INTENTIONS**

- Do you think MTC will effectively manage the medicines selections in the future?

**I. GOALS**

- Considering all the things you have to do when you are performing activities of the MTC how important is selecting medicines for the MTC? Why?

**J. MEMORY, ATTENTION AND DECISION PROCESSES**

- What thought processes might guide your decision to select appropriate antibacterials for the formulary?

Prompts: What goes through your mind? Is it a routine part of the job or is it something you need to take time to think about?

**K. ENVIRONMENTAL CONTEXT AND RESOURCES**

- What factors in your working environment influence the MTC optimizing antibacterials in the hospital?

Prompts: the existence of trained staff, too busy, don’t know how to, other priorities

**L. SOCIAL INFLUENCES**

- Would other members of your team influence how you select medicines for the hospitals?

**Prompt**: what about the [GP, Practice Nurse, DSN, CMHN, Psychiatrist] - do you discuss the selection of medicines with them? How might those discussions affect your decision on the selection of medicines?

N. **BEHAVIORAL REGULATION**

- If you wanted to change the way the MTC promotes optimal antibacterial use, how would you do this?

O. **PATIENT POPULATION**

- How do you engage with prescribers to actively prescribe selected antibacterials on the formulary list?

Close: That’s all the questions I have for you, has anything occurred to you about this topic during the interview that we haven’t discussed?

Thank you for taking part.
