# Supplementary material for: The role of medicines and therapeutics committees structure in supporting optimal antibacterial use in hospitals in Uganda: A mixed method study
Source: PLoS One. 2024 Jan 19;19(1):e0289851. doi: 10.1371/journal.pone.0289851 (PMC10798471; doi:10.1371/journal.pone.0289851)
Supplement: S3 File — (DOCX) [file pone.0289851.s003.docx]

**The role of medicines and therapeutics committees structure in supporting optimal antibacterial use in hospitals in Uganda: A mixed method study**

# TRANSCRIPTS OF INTERVIEWS

**Pharmacist KI #01**

Age: 46

Sex: Male

Department: Pharmacy

Current position: Senior Pharmacist

Years in practice: 21

Duration of interview: 43.23 minutes Gender: Male

**Interviewer: How long have you had medicines and therapeutics committees (MTC)?**

**Respondent:** Now, Six years

**Interviewer: What is your role in MTC?**

**Respondent:** I am a secretary in the MTC and have been performing this role since it was established

**Interviewer: who are the members that make up your MTC?**

**Respondent:** We have representatives from all the mainstream departments, that is, medicine, surgery, obstetrics and gynaecology, and Pediatrics; then we also have members from the laboratory, then the pharmacy department, then from the administration and also from the store's department.

**Interviewer: How does the MTC choose its membership?**

**Respondent:** Over eight years ago, the MTC was dissolved because it was not operational. In 2014, the administration reappointed new members. It was decided not to select senior medical consultants who were nowhere or never showed up for the meeting due to other commitments. We now value the interest and dedication of a member over seniority in a medical position when choosing new members.

**Interviewer: what levels of qualification are required for members of MTC?**

**Respondent:** We welcome every member of staff as long as they are committed and interested in performing the MTC's role. Senior clinicians are no longer the main target for membership. All junior medical officers, pharmacists, and nurses are eligible for membership. We now have clinical officers, senior nurses and midwives. Then, we have a principal laboratory technologist and a principal psychiatric officer on the committee; these people can attend the meeting.

.

**Interviewer: What are the main functions of the MTC?**

**Respondent:** One main function of the MTC is the rational use of medicines to improve clinical outcomes and reduce hospital wastage, ensuring the appropriate use of antibiotics to mitigate antimicrobial resistance development. Then, the other key role is in the supply chain function. In the supply chain, we're talking about the supply chain cycle from selection, procurement, distribution, storage, and use. Then, we also look at issues of pharmacovigilance, where healthcare workers identify and report on adverse drug reactions. Rational use of antibiotics through antimicrobial stewardship is a key function. Then another issue, which would have come under appropriate medicine use because of the level of its importance, we put it as a block, and that is antimicrobial stewardship,

**Interviewer:** **What skills are needed to perform the functions you mentioned?**

**Respondent:** Our membership has people with different types of professional training. Yeah. For example, we have clinicians specialising in infectious diseases. And that is that TB, we have one in TB, we have one from HIV. Now, those guys participate a lot in the antimicrobial stewardship committee. Pharmacists participate a lot in the pharmacovigilance Committee because they are concerned with identifying and communicating adverse drug reactions.

**Interviewer: what criteria guide your selection of medicines or for the formulary as MTC in this hospital? Is there a criterion you try to follow?**

**Respondent:** In the management of the formulary, the budget is, without a doubt, the most significant concern.  It carries the utmost weight and significance in determining which antibacterials we shall select or procure. MTC meets to figure out which antibacterials are expensive and reasonably priced to keep the budget under control and keep expenditures to a minimum. Then, we also look at the spectrum of activity. Then, we also consider current morbidities. Normally on morbidity, the Ministry of Health makes one report for all the hospitals in the country, and this is categorised for different regions. In that case, we need to construct a morbidity report of our own we have our own. We can also extract reports for our hospital from DHIS2. Then, You know that there are two categories of very important people: children and mothers. For example, the choice antibiotic is ampicillin. We need many metronidazole injections for the mothers when they have been censored, something like that after delivery. So we do not want those categories of people to lack medicines. Because we are trying so much to reduce infant mortality, maternal mortality, neonatal mortality,

**Interviewer:** Can you share some particular goals you focus on when selecting medicines?

**Respondent:** We focus on several goals, one of which is to reduce maternal and childhood mortality. We are trying to minimise stockouts in mothers and children of essential medicines in the hospitals. We also ensure we have lifesaving medicines for all categories of patients.

**Interviewer: How easy is it to implement some of the mentioned roles?**

**Respondent:** I can say that it needs much patience, especially from the secretariat because it's the main driver of the medicines and therapeutics committee, so because the roles are many, To ensure the MTC's effectiveness, we established subcommittees. Subcommittees then carry out their mandates for the MTC. So, if your work is antimicrobial stewardship, that is the target role. So that you just report to the MTC during its meeting.

**Interviewers: What type of skill or competencies are needed to perform the functions of MTC? Respondent:** Yeah, that is very important because let me first talk about the secretariat as medicine and therapeutics committee for the hospital. And the hospital has only one pharmacist. That is quite, quite tough for that secretary to follow up. If we had several pharmacists, in that case, we would have a secretary in each of those subcommittees, and we even have a committee for appropriate medicine use because that one can look at other medicines, and then the stewardship committee looks at how to use available antibiotics appropriately used to reduce antibiotic resistance. The committee has conducted interventions to improve antibiotic use. So you have secretaries who are pharmacists in each of those. Then still the membership of the MTC, you see, we needed people like paediatricians and gynaecologists, but I don't know whether it's only at this hospital, but those people tend to be very busy and do not have time.

**Interviewer: How often do you members of the MTC meet?**

**Respondent: As a committee, we operate under certain terms of reference. We meet once every two months, and then the subcommittee meets once every month since they are carrying out these responsibilities.**

**Interviewer:** **What are the major topics of discussion?**

Respondent: In most instances, we have challenges associated with the supply chain regarding the procurement strategy, the ordering, the distribution, and the use of medicines. Antibiotic-related issues like antibiotic resistance have received significant attention this year.

**Interviewer: What are the major challenges you face as MTC?**

**Respondent:** One of the problems we have is that some of our members have more than one job task at the hospital. This makes it hard to get people to the meetings. People's schedules can also make it hard to follow up on how the MTC activities are done. There aren't enough people to do the work, which is a big problem; it is a huge issue. Furthermore, there is no clear facilitation, either. Some people come to meetings hoping to be motivated; they only receive a soda during a meeting as a token of appreciation. Another problem is that members do not want to do anything apart from their core duties.

**Interviewer**: **How can the MTC remedy these challenges?**

**Respondent:** In order to keep up with the growing number of committees, the hospital must set aside a budget for them since, due to limited resources available, it's challenging to allocate each committee a budget. However, the committee budget would be used to facilitate meetings and keep members motivated. It would be used to provide refreshments like soda and snacks. If the hospital's financial situation improves, members may get an allowance.

**Interviewer: Has the MTC investigated medicine use problems?**

**Respondent:** We have had an issue with gentamicin by now. We should not have any stock in the hospital for the last six months or more. We had several adverse drug reactions with gentamicin. Our subcommittee on pharmacovigilance came up and investigated it. We picked many reports and submitted them to the National Drug Authority. Surprisingly, other regions were also reported, so NDA made many recalls for all batches of gentamicin from different parts of the country. Until now, National Medical Stores, which is our supplier, had never gotten us a new supplier. And normally, making a new contract takes time. We had an extensive stock of gentamicin in the whole country. If you find gentamicin in a particular hospital, it has never been taken back to NDA.

**Interviewer:** Has the issue of gentamicin been solved?

**Respondent:**  No, National Medical Stores has not yet gotten a supplier that the National Drug Authority approves.

**Interviewer:** Have the criteria solved antibiotic stockouts reserved for mothers or children?

**Respondents:** Yes, we no longer have stock out. If you hear of a stock out, it is a manufactured stock out. Now, the only challenge with antibiotics is the criteria we use to select the few that can be accepted by everyone or satisfy every patient's needs.

**Interviewer:** How would you compare your hospital with other hospitals in the form of fighting stockouts? Are you doing better?

**Respondent:** First, this facility is the busiest regional referral hospital. Yes, it is the most efficient regional hospital in the country and holds the number one spot in all categories, including surgery, mothers' maternity care, newborn babies,  and even referrals of neonates and sickle cell cases. We have the biggest sickle cell clinic with more than one thousand children who can access hydroxyurea treatment for sickle cell disease.   If you compare our hospital to others, you will see that we have done an excellent job of preventing stockouts. Okay, I  can comfortably say that we have somehow succeeded because, over the past five years, the government has doubled our budget for medicines.

**Interviewer:** What is your comment on antibiotic use in the entire hospital? Would you say it is appropriate? Are they trying to use them rationally?

**Respondent:** Our doctors disagree with the antibiotics we choose because they prescribe antibiotics off the formulary list. Those who prescribe antibiotics on the formulary abuse them often, causing resistance to develop. Getting a culture and sensitivity report is difficult, making it hard to know which bacterial pathogens are sensitive.

**Interviewer:** What do you think drives prescribers' non-formulary writing?

**Respondent:** it is staff shortage and attitude. Staff don't believe in antibiotics we have on the formulary list. They look at a patient and think that this patient will only benefit from a superior antibiotic. Most of these antibiotics are non-formulary.

**Interviewer:** What are the drivers of this seemingly inappropriate antibiotic use?

**Respondent:** the biggest driver is the drug promoters. They lure prescribers to prescribe antibiotics from the formulary and influence antimicrobial resistance. Also, resistance may not be from only the clinicians, and if they have been prescribed, ampicillin is supposed to be given for 6 hours. We have one nurse covering all night from bed number one to bed number last, which can take 2 hours. Most ampicillin is because they are not given the drugs at the right time.

**Interviewer:** Okay. How have the MTC tried to address this problem of inappropriate antibiotic use?

**Respondent:** we have done CMEs on antibiotics themselves. We have controlled drug promoters from accessing prescribers, though it is still a big challenge. We are ensuring that they come at a specific time. If they want to see the prescribers, let them do a CMEs, not meeting one on one. So if they can do that, then we also ensure that they give a good CME, which is not a biased CME. We plan to produce an antibiogram with the stewardship committee to look at all the ward resistance patterns and then disseminate the results quarterly.

**Interviewer:** **How confident are you about MTC solving inappropriate antibiotic use problems shortly? Do you feel you're going in the right direction to curbing antibiotic resistance?**

**Respondent:** MTC is growing more extensive, but the government doesn't have the will to employ more workers, so clinicians are overwhelmed. If we try to bring a CME, everyone is too busy seeing patients. The secretariat itself is so busy, and we don't have the staff. You can't be everywhere. And if you do that, you break down.

So, the MTC can control the problem to a lesser extent, 30% or less. Then, we also need to improve financing for medicines because, as I've told you, we have very few carefully selected lists of antibiotics. But if we could have two or three second-line antibiotics, we could enforce it on our clinicians that these are the two-three antibiotics you can prescribe, but that comes in with the increasing budget for medicines so that you don't bring so few serve so few patients. You don't complete doses, which increases the problem of resistance. So, if we can improve the financing for medicine, we can have more support from the hospital's leadership. Yeah, just to keep the activities of the MTC, something like that. Yeah, some of those things can be done, then, of course, also the infrastructural arrangement of the hospital. For example, it's an extensive department for obstetrics and gynaecology. Can we have a fully-fledged pharmacy that can serve all the patients there, and even the people sitting there can do pharmaceutical care? And follow up on all the patients. We could even have MTCs specific to departments so that they can handle their issues. Yeah, but as we talk now, everything's so ambiguous.

**Interviewer: If a hospital didn't have an MTC, do you feel it would be affected severely in terms of medicines wastage and, others, inventory management?**

**Respondent:** Without it, there will be a lot of blame game here and there. You will put much work into just a few individuals, and all will be given the blame for all the problems in the hospital.

Yes, because the MTC does a lot of supervisory roles. Then, while in this role, the MTC does a lot to enforce appropriate medicines. Yeah. But if the MTC is not there, you need to get good technical people to manage medicines.

**Interviewer: In your opinion, Are you serving your patients with the MTC's existence compared to when it was not there?**

**Respondent:** I would say that to the extent of 30%. Like because of the challenges I've told you about in the MTC. To an extent of 30%, We are serving our patients better. Yeah. Because we shall say no in those meetings, let's remove this item. Let's include this item. So let's increase the quantities of these items. So at the end of the day, the patients benefit.

**Interviewer. Okay. What do you think might happen if certain functions that you've mentioned some key functions, like pharmacovigilance, are not performed?**

**Respondent:** If pharmacovigilance is not carried out, allow me to provide you with an illustration of a medication known as bupivacaine used for anaesthesia. It is true that during the labour and delivery of many mothers, we had many setbacks in administering the first spinal injections since there was no response. We had to administer the second, third, and fourth injections because the initial injection failed to work, but the mothers remained with no response. So, we needed to report this occurrence to the pharmacovigilance committee. And for some reason, when we reported the issue to National Drug Authorities, they said this is just one report from a single facility, and I do not think we shall utilise this one report to make a decision. As a result, I was forced to organise my colleagues who worked at other regional hospitals and ask them to watch this medicine kindly. They discovered that they had the same problem but chose to ignore it. When they finally did report it, the entire batch was recalled.

**Respondent:** MTCS now has increased receiving alerts on medicines used in the hospital. Imagine a patient receiving ceftriaxone in the ward for 5,10,11 days and is not improving. A culture and sensitivity test may be required for such a patient; then, after appropriate medicines are given, which takes 3,5 days and the patient is well. So, those roles of MTC, if not performed, will severely affect the patient.

**Interviewer: What about the hospital, is there improvement in their expenditures like reduction of wastages, Inventory Management? Has the MTC improved that?**

**Respondents:** Without MTC, our hospital would be in great difficulty, and there would be widespread stockouts. I can assert that the hospital alerts on stockouts have decreased since we developed MTC. They have guided well wards on medicines use and avoiding wastages. MTCs are successfully controlling medicines budgets, which has led to an increase in the number of drugs available in hospitals.

**Interviewer: How has the MTC reduced distress in the supply of medicines like antibiotics?**

**Respondent:  the** majority of clinicians now recognise the importance of the MTC's role. They have constant access to medical supplies and medications, including antibiotics. They are provided with information about the appropriate usage of pharmaceuticals.

**Interviewer: Okay. What motivates you as a member of MTC to continue serving and serving at this committee?**

**Respondent:** I even felt like resigning because of the challenges. You could get data on morbidities, and the government looks at it as if they do not consider that. You have 100 patients; they give you money to treat only ten patients. It was discouraging, but continuous use of the data and lobbying with the government has had some fruit over time.

**Interviewer: How has the MTC used data generated for proper governance?**

Respondent: normally, when these ministry officials come to the hospital, you can have, like, some members of the MTC meet those guys and maybe people from national medical stores, then we also produce reports as MTC and forward, there are complaints which we have made, but we succeeded that one year ago when at least they realised that Mbale needs a better consideration when it comes to financing for medicines so what motivates me is that finally, at least all the struggles we have had, sometimes at least now people are trying to listen

**Interviewer: What activities do you think you will do much better from the experience you are gaining, and what do you hope to do much better in the future? Maybe in the next five years?**

**Respondent:** One of the key achievements of the MTC is infection control. Because whichever point you go to in this hospital, you'll find hand disinfectants and antiseptics, and that one has reduced scepticism in the hospital. Now, we want to scale up access to sanitisers to patients and their attendants in different wards. We already have sanitisers in the neonatal unit because of the sensitivity of neonates, attendants, and the mothers in that unit who have to use them.

**Interviewer:** where do you get these sanitisers, if I may ask? Are they procured?

**Respondent:** With the help of the supply chain function of the MTC, We decided that hand sanitisers are very important, so as a hospital, we have appropriated about 50 Million from our medicines budget for only hand sanitisers. And now we are looking to raise that to about 75 million.

**Interviewer:** How has MTC supported your infection control in this hospital? Do you feel it is worth it?

**Respondent:** We know that a doctor picks one strain from one patient to another. If you are the last patient, imagine the types of infection you will get. So, with all the efforts on infection control introduced by MTC, the number of doctors, clinicians, and nurses infecting patients has diminished.

**Interviewer:** Where are you going to focus most in future as MTC?

**Respondent:** One of the subcommittees we shall give more attention to is antimicrobial stewardship. We want to boost that subcommittee and have many activities being carried out. We also want to carry out research in antimicrobial stewardship. We want to look at the different levels of infection of microbial contamination on other surfaces in the hospital and wards and then look at what we can do to reduce contamination levels.

**Interviewer:** What enabling factors in your working environment have influenced performing MTC?

**Respondent:** The appreciation and establishment of a policy to have MTC in every health facility invigorated us. It was a big push in the right direction. The Ministry takes MTC so seriously that it requires all hospitals to report on activities.

**Interviewer:** What about the team members who have worked with you? Are they supportive? Have they supported you enough?

**Respondent:** the current committee is very supportive because they come for meetings, but they keep complaining that they can't have a special allowance. Hmmm...I was told that the motivation we give to health workers is a snack or a drink. You know promotion in government is very competitive. I am not sure they follow merit. There are so many issues that I don't want to talk about. But of course, the chairperson, if she performs well, can be recommended, and even the secretary can also be recommended for promotion. But even then, the promotional…, can I say the career path for a pharmacist in the regional hospital senior pharmacist is the highest position.

**Interviewer:** **What is the influence of hospital members on your activities? Do you feel these members are beginning to come on board seeing you perform all these activities? Are they beginning to acknowledge your performance?**

**Respondent:** let me just say this: there was a time when the budget for medicines was so low that no one was interested in the MTC. They were like, what are you going to do? What are you going to monitor? There were always no medicines in the hospital. You are telling us you will do a procurement plan, so what? You will plan for very few items that will not even serve the hospital. So we had that history in that so many people did not want to be involved in the MTC because of the severe conditions. Before the MTC, the budget was minimal, stock out was so much, and most hospital staff were not motivated. You can call for a staff meeting or a CME, but they didn't want to get involved at one point. People had a bias. They even tended that, hmm, like they had given up and imagined a situation where there were no cannulas, syringes, or gloves.

Of course, by the time you do not have syringes, you do not have the drugs. That is a huge demotivating factor. You do your activities and want to promote appropriate medicine use, but many stockouts exist. Yeah! We give information in the staff meetings, that's the general staff meeting. Then we also give information in the senior staff meeting, which is done every morning. I think you have heard of that meeting.

**Interviewer: are you satisfied so far with the work you have done as the MTC in the hospital? Are you feeling a little bit that there is somewhere you're going?**

**Respondent:** As MTC, I am satisfied to the extent of about 30% because members tend to give much bias concerning MTC activities to the secretariat. You understand. They don't emphasise whether it is about supply chain and procurement planning. They want to push it over. One, the secretariat should be boosted in terms of staffing. Then, let us think of some mechanism to motivate these people because they think that the secretariat is using the MTC to achieve their goals. (Laughs) un yet the motivation that the members get is not different.

If you get a snack in a meeting, that is all; there is nothing more but they think that the secretariat is using those meetings or whatever interventions for their benefit, so they are fully satisfied that we are working to an extent.

Interviewer: So could the solution be that you coopt some members to the secretariat such that there is a balance in between?

Respondent: We have done that, but they are not sometimes very technical. We want somebody who is coopted but also benefits from it.

Interviewer: you want a technical person to the secretariat; which qualification or position?

Respondent: it should be a pharmacist at any level.

Interviewer: So I would want to get your comment, maybe because we have to end our interview. How do you feel about this interview? Has it exhausted everything you would have to say about the MTC work you have been doing? Do you have any other feedback you would want to give us so far from what we have discussed? Do you have any supporting documents you could email us to go through to understand what is going on, like a small report about you?

Respondent: I think the interview has been exhaustive on several issues, though, at one point, it was biased on antimicrobials

**Pharmacist KI #02**

Age: 52

Sex: Male

Department: Pharmacy

Current position: Senior Pharmacist

Years in practice: 26

Duration of interview: 33.23 minutes

Gender: Male

Masters in procurement and supply chain

**Interviewer: How long have you had medicines and therapeutics committees (MTC)?**

Respondent: The MTC in Jinja was established seven years ago

**Interviewer: What is your role in MTC?**

**Respondent:** I am a secretary in the MTC and have been performing. I was a secretary in my previous hospitals, I am now a secretary in this hospital.

**Interviewer: Who are the members that make up your MTC?**

**Respondent: W**e follow the WHO guidelines when selecting the MTC membership. Ideally, they need all the major specialities from medicine, paediatrics, surgery, obstetrics, and pharmacy. The head nurse, the laboratory head, and then someone in administration must be there. Each MTC has different needs, so membership varies. Our MTC has all major clinical specialities in the hospital. Yes, we have a paediatrician, surgeon, physician, someone from administration, biostatistician.. The physician is the chairman, and the pharmacist is the secretary.

**Interviewer:** What criteria are used in the selection of medicines?

**Respondent:** I remember an experience in previous years. We had an anesthesiologist for the first time in our hospital. He requested that I, as the secretary of MTC, include a drug for his unit. Then, we included it, and when it was supplied, she went to Germany for a course. That medicine ended up expiring because no other person could use it. This made us decide on selection criteria where no single specialist would be the beneficiary, but all the other specialities should be able to use the medicine or benefit most patients. Currently, our criteria are based on the user department's needs. At the beginning of every financial year, we make the procurement plan. In this procurement plan, all departments have their wish list of what they need. And then, for antibiotics especially, we are active. We do culture and sensitivity tests and develop a sensitivity pattern for the antibiotics in our hospital. Procurement planning is one of the biggest things I do. So, the lab made a presentation on our sensitivity pattern. Now, the members select based on that. Our hospital has proven that ampicillin is almost useless, as I talk to you now. ^[[1]](#endnote-1)^

**Interviewer: How have the criteria of medicine selection helped increase the availability and use of antibiotics?**

Respondent: True, it is helping, but of course, you know we have a subcommittee, the Antimicrobial Stewardship Committee. We call it AMS, whose goal is to promote the appropriate use of antibiotics and minimise the development of antibacterial resistance. AMS committee adopted the WHO categorisation of antibiotics in our hospitals into three groups. The first group is called access because everybody prescribes them from clinical officers, medical officers, and special grades. Then, there is a second group. That second group is slightly restricted to senior doctors, not clinical officers. We have two antibiotics in the third group called to reserve: Meropenem and Piperacillin/Tazobactum. They have used this categorisation to introduce other AMS interventions like Pre-authorisation and restriction to reduce misuse and wastage of reserved antibiotics.

**Interviewer: What interventions have the antimicrobial stewardship committee instituted**

Respondent: We introduced pre-authorisation or restriction as an antimicrobial stewardship strategy where only medical specialists would access or approve reserved third-line antibiotics. Most medical officers were against this strategy since they were left out of the use of such antibiotics, but generally, this improved treatment outcomes for children with severe infections. So that one is reserved. The MTC required before the use of the reserved antibiotics is that the attending specialist should also have a report of culture and sensitivity testing indicating the sensitivity of the reserved antibiotic*.*

**Interviewer: How has the MTC guided the use of these reserved antibiotics, more so third-line antibiotics?**

**Respondent:** Yes! You know what we have is an SOP; not everyone will prescribe Meropenem unless you have the recommended level of speciality as recommended by our SOP. We also give a lot of CME to guide staff on using second and third-line antibiotics in our hospitals. We also want to do CME to create awareness of antibiotics.

**Interviewer: What criteria has the MTC used to select antibiotics**

**Respondent**: Yes, and we do what is called ABC analysis. In our MTC, we analyse drug consumption data twice a year. The report will show us how we use antibiotics in the wards. The last report showed we stood at 78%, which is not good yet. We have just analysed data from one of our last meetings of MTC, which I can share with you. We discovered the use of ofloxacin was 2.1%, and Cipro and Septrin were 2.6% and 2.6%. In this ABC analysis, you cannot use ofloxacin, which is the third line to Cipro and Septrin. That is how we do our stewardship: we get data, analyse where the problem is, and then come to the meeting.

**Interviewer: What interventions have you put in place to optimise antibacterial use in this hospital?**

Respondent: We have already selected a list of interventions to adopt from the government. We juggle within that list. Mostly, we conduct culture and sensitivity for all antibiotics in this hospital. If the sensitivity of the antibiotic has gone so low, we do not need that antibiotic in our hospital. Unlike other hospitals where doctors perceive that some antibiotics are not working without proof, in this hospital, we have to conduct regular culture and sensitivity testing, and from those reports, we can decide as MTC.

**Interviewer: How has this routine culture and sensitivity intervention supported members using antibiotics on the formulary?**

**Respondent:** We have an outstanding paediatrics team that appreciates formulary antibiotics for children. Though we have never conducted some assessment to confirm that adherence to prescribing on formulary has increased, clinicians have increased requests for antibiotics in their paediatric ward.

**Interviewer: Are there any skills or expertise needed to perform MTC functions like rational antibiotic use and pharmacovigilance? What is your comment?**

**Respondent:** Well, I think the skills are generally needed, but the first skill you need is simply loving what you do. There are so many people with better skills than me, even in education and exposure, but they are not motivated to join MTC. The first thing we need is your time. I can tell you that you will get people to help you when you start. Even when you reach a dead-end, there will be people who will help you as MTC. As I talk now, when we just started our MTC, the skill set was really low. People did not have skills. However, we connected with the London School of Tropical Medicine, which trained members and got support and training twice with Makerere University. They trained us on implementing antimicrobial stewardship interventions.

**Interviewer: How confident are you that your hospital MTC can solve inappropriate antibiotic use in children under 5?**

Respondent: We have had successes because a paediatrician has decided to institute antimicrobial stewardship interventions in his ward. The interest of the paediatrician in MTC works changed my perception of who should form membership. I look at the people who have interests, much as the recommendations from WHO say heads of departments. I don't care if the junior is more interested than his seniors, than he is eligible for membership in MTC. We work with the paediatric on implementing antimicrobial stewardship. He consults the MTC on every intervention he implements. a kid came in with a resistant infection to all the important antibiotics, including the third line like Meropenem. We conducted culture and sensitivity to Clotrimoxazole. The kid was given cotrimoxazole and discharged

**Interviewer: Are you optimistic that the MTC will continue doing good work in the future?**

Respondent: We would have been far if we were not interrupted by the transfer of our members. We have just replaced some of those members. By the way, we are also doing consumption, and we are trying to follow up on the consumption of antibiotics. I am also doing a small study comparing consumption in Jinja and Mbarara. The question I want to answer is the high consumption of antibiotics. Does it necessarily translate to resistance to that antibiotic? That is what I am doing.

**Interviewer: What do you think would happen if the MTC did not attend to the major medical issues/problems you are experiencing in the hospital?**

Respondent: it would be a mess. First of all, there is theft in these hospitals, theft of medicines, and the antibiotics are not spared. Now, if the MTC is not active, a lot of wastage and theft of medicines would occur. The existence of MTC has reduced theft and also promoted the improvement of medicines use. Whenever there is an inappropriate use of medicine in a prescription, the member of MTC will follow it and discuss it with the prescriber.

In most cases, clinical officers write antibiotics when it is not necessary. So, the MTC keeps people on their toes. Someone who is going to come up and follow this.

**Interviewer: What changes have clinicians experienced ever since the MTC was established?**

**Respondent:** In 2017, the medicines used in the hospital were a mess. A clinician would prescribe any medication he wanted off or on the formulary list. So the MTC arranged with IDI Makerere, and we took them one week of training on antibiotic prescribing to curb the practice of prescribing off the formulary list. However, you know, the thing is we are very funny people. After training, antibiotic prescribing improved for a short while, and people relapsed. To avoid this, training should be continuous, or there should be follow-ups because it improved after that. I was doing an assessment before and after, which came up to 80% proper prescription. But I have not been doing that now, and it may have gone down. Another weakness is that people associate me with the MTC. SO people just fear when my presence and tend to do the right thing.

**Interviewer: How could you improve on that so that they can continue doing the right thing even when you are away?**

Respondent: Clinicians know the right things but want to do the wrong things in human nature. So people tend to do the good stuff when they see a member of MTC around, especially the clinical officers and interns. This has been my challenge. The MTC wants to be de-touched from individual members to a system, but that has been a challenge.

**Interviewer: What has always motivated you to remain an active member to perform activities of the MTC?**

**Respondent**: When you give me something to do, and I accept, I will commit and do it to perfection. I make a decision. Once you tell me to do this thing for me, I want to do it properly. So it is some bit of self-motivation. As a pharmacist, even without the title of secretary MTC, it is my role to see that medicines are correctly used. That is at the back of my mind. So once The other issue is, as a pharmacist, whatever happens in medicines, I answer, I am answerable. I should be able to explain what I have control of. In this hospital, my primary responsibility is medicines. Just like the auditors are around, in some cases, when the auditors come, people run away. For me, I am assured of what I do. When they ask for this, I look for it and explain it when there is a mistake. There is no deliberate mistake, and I make mistakes like anyone else. So when these guys came from the Ministry of Finance ministry, even like yesterday, they came when I was already going home. I just parked the car and came and gave them the documents they needed. In other places, guys try not to be around.

**Interviewer: what functions do you think the MTC will perform much better in the future? Is there a function you feel you are going to excel in in the future?**

Respondent: I think the main function of the MTC shall remain medicine selection. If you select wrongly, then most of the things will be wrong. Maybe the other thing is to be on your toes because you do the best procurement planning as an MTC. If you do not follow up, the same good procurement planning is poorly managed. I can explain to you that. The issue with the health workers' behaviour is that they can make perfect procurement plans; the paediatrician says MTC, we must have Xpen. It is an antibiotic used in paediatrics and others like ampicillin. Because the nurses in the ward are few, you find one nurse in the award, and she has to administer to over 30 children Xpen. I started seeing that the Xpen in the ward was expiring, the five mls injections were accumulating, and then the 10mls were running out. So we went out as MTC to investigate the issue. So interesting: the doctor prescribes Xpen 6 hourly and goes home, and then the nurse gives ceftriaxone once instead. The doctor does not know. He thinks the patient improved because it was Xpen. We realised why Xpen was wasting time on the wards and why nurses were not administering it. The nurse administering to 30 children Xpen 6 hourly necessitated much work. For convenience, ceftriaxone is given once. As MTC, we realised that conducting a root cause analysis enabled us to know the source of the problem of Xpen wasting in our hospital. The selection of medicines is critical and must be incorporated well in procurement planning.

**Interviewer: How has the antimicrobial stewardship committee worked on the issue of use and misuse of antibiotics?**

Respondent: That's a huge issue. I can further tell you what we found out in one of our medicines investigations. We found out that patients come to the orthopaedic ward when they are rotten. They come so bad: the nursing system is a bit poor, the ward is dirty, people come late, and the orthopaedic surgeon immediately goes for the third line. So what we came up with, we are not going to accept until they provide culture and sensitivity and the cleanliness of the ward has improved. This was discussed. That is use

**Interviewer: What thought process has guided your decision when prioritising the activities of the MTC?**

Respondent: Work plan. What I do is I come up with a work plan as a person, and I bring it to the deanery to discuss. Then, they discuss and come up with the timelines. So they will tell me," Olum, I think we will need this one," or they will tell me, "No! This one is not the most important, the most important, so be this." Things like that. So, we work against a work plan.

**Interviewer: What factors in the working environment are influencing the MTC to perform better, or what is inhibiting it from performing to its peak?**

Respondent: The thing is, the administrative support. The director and administration have given the MTC the go-ahead on matters of medicines management. We always share a report with the director and administration on our progress and what kind of support we need. The other issue is small things like simple funding. We were doing well when IDI gave us 200,000/= for snacks and things like that each time we held a meeting. These snacks were so motivating. If you call these consultants like that, and they go for a meeting without any soda, the next time, the number will drop by half. So those simple things like refreshments during sessions. And then sometimes, when you train them, especially when you take them outside the facility where they stay, these are public facilities, they get motivated when they are given some small-small things. For the training we had with the London school of hygiene, we took them across the bridge in another district, and that was Buikwe district, so because it was another district,, they had to pay them something, which So those simple motivations are very important.

**Interviewer: What is the influence of hospital members generally concerning the MTC activities?**

**Respondent:** Uhm, naturally, when you do certain things, some people will support you, and some will not support you. I started an inpatient pharmacy. What we used to do was issue drugs from the stores to the wards in bulk. The wards became mini-stores. The nurses would steal these medicines. Or they would tell you, that this drug is not there, then they tell the patient to give money, and then they go back and provide the same drug we have issued to the patient. So what I did, I stopped that. So, we issue only to the inpatient pharmacy. Naturally, these people on the wards benefiting from the ward supplies are unhappy with MTC because I cut their lifeline. However, MTC has made some people happy because patients come and take what they are supposed to get. Even some nurses are happy because they say, "No, we used to feel funny, but now people are improving and going away", so the ones who don't like MTC are clinical officers. I don't know if clinical officers are born from the same mother. They always want to do wrong things, so when confronted by MTC, they fear someone who corrects them.

**Interviewer: How could you influence senior consultants to participate in MTC activities?**

Respondent: I have done that; incidentally, another person is doing it much better. Being a consultant, he has shared his previous role in MTC and encouraged colleagues to influence fellow consultants on the quality of patient delivery. They are very good people.

I don't think that the government people should leave things like health education just for private-sector pharmaceutical companies like that because the quality of the clinicians we see coming up is not good. It's the responsibility of the senior consultants to mentor these young doctors. I didn't only join MTC to conduct CME when we got a problem. We want to engage everyone to be accountable.

We conducted a focus group discussion with clinical officers to get their views on the use of medicines. It was so good. The MTC wanted to introduce interventions to curb the irrational use of antimicrobials like antimalarials among clinical officers. The government policy was test-and-treat, and the first line is Coartem, but they were doing different things they were not testing. So, I just said no! I called all clinical officers with a physician. I showed them their prescription. So they were shocked, so I asked why this happened. So, I got good reports and feedback from the focus group discussion. We opened a mini lab and made RDT available around so they could do RDTs. Also, I removed Artesunate from there. Artesunate is only obtained from the inpatient pharmacy when you result from BS, not RDT. The senior consultant was so motivated and realised he had to play a role in the MTC.

**Interviewer: What outcomes were generated from this intervention of a mini-lab?**

**Respondents:** We were using 12000 doses of antimalaria in 2 months. Introducing \mini-labs reduced it to 3,000 doses in 2 months from the 12000 doses you would use and not finish the two months. So that is the importance of the MTC in minimising medicine waste and cutting costs for the hospital.

**Interviewer: So any challenges and what you suggest, how can what you are facing be improved in the MTC?**

Response: Aaah, the bigger challenge I have already alluded to is the MTC being tagged to a man. It should not be tagged to a man, and it should be a hospital thing. The next challenge, as I said, is administrative support. We rely on mostly NGOs. If the NGOs are not there, it seems our facilities cannot support them, at least in terms of small-small funding. Ideally, according to the Ministry, it is supposed to be part of… That is one standing committee of the hospital. It is supposed to have been funded. That is the next challenge. Maybe the other challenge is general funding, which we do not get. All the drugs we get are not exactly how much we would have wanted. But because we have a budgetary ceiling, we have to juggle within to make the procurement plan so that you have maximum from your little. So funding is that problem.

Interviewer: thank you so much because I have learnt a lot

**Pharmacist KI #03**

Age: 37

Sex: Male

Department: Pharmacy

Current position: Senior Pharmacist

Years in practice: 13

Duration of interview: 30 minutes Gender: Male

**Interviewer: How long have you had medicines and therapeutics committees (MTC)?**

**Respondent:** Now, Six years

**Interviewer: What is your role in MTC?**

**Respondent:** I am the chairperson of the MTC. It is over two years since I was elected to the position.

**Interviewer: How many other members are on the committee, and which department do they come from?**

**Respondent:** The administration appointed seven members, but two members retired. We hope to have replacements after the expiry of the three-year contracts because the hospital director on contract appoints these members. So after that, members shall again be relieved of duty or reappointed with new contracts depending on performance....

**Interviewer: How does the MTC choose its membership?**

**Respondent:** We modified membership according to the WHO guidelines. We have representatives from all major medical wards: gynaecology, surgery, internal medicine, and paediatrics. Then we go on to smaller areas like psychiatry, dental or eye, OPD, and community department. Then we also have the stores. The pharmacy itself has to be represented. Then, we also coopt all the pharmacy interns for mentorship. Then radiology because of its uniqueness. We also had the administration. Yeah, they should also be there because, you know, you can't run apart from the support from the administration. The director somehow, but not appointed, for we want a wholesome representation.

**Interviewer: How often do you members of the MTC meet?**

**Interviewer: what levels of qualification are required for members of MTC?**

Respondent: Yeah, we do not emphasise the qualifications. However, what we look at is how active you are. Nevertheless, then we also look at the units of representation. That is why our numbers are big; they should have been around 15 or less. But because

**Interviewer: Well represented indeed, what are the subcommittees you're having?**

**Respondent**: We have several sub-committees that perform the functions of the MTC. We used to conduct operational research, so we had the research committee. It is not very active right now, but we also have an antimicrobial stewardship committee in charge of the sensible use of antibiotics at our hospital. We also have a pharmacovigilance committee and a records committee, which we used to have. So, we had certain committees at the time because we were looking at record problems. We have a supply chain or logistics committee that looks into drug supply concerns, such as purchase, supply, storage, distribution, and usage.  We create the committee when needed. The subcommittees undertake the activities and then report back to the larger committee..

**Interviewer: What are the main functions of the MTC?**

**Interviewer: what criteria guide your selection of medicines or for the formulary as MTC in this hospital? Is there a criterion you try to follow?**

**Respondent**: Wow, that is a good one. As I mentioned earlier, our supply chain or logistics committee is responsible for managing the supply of medicines on our formulary list. The logistics committee and the pharmacy department prepare the consumption data for the procurement planning, though it is not the best way, that's what they do. We try to see what consumption and morbidity are in the hospital. We also discuss new requests for adding or deleting some medicines as MTC. So, we meet with National medical stores on the entire procurement plan.

We, as MTC, are not practically involved in the selection at the national level, but they have a template already formulated. In that template, they assess their advance in terms of payment that is factored in there. The challenge is money is already put there, too, you can't go beyond these, you have to fit your needs within this.

**Interviewer: Has the MTC investigated medicines use problems? So I have been to various wards; gentamicin seems to be a medicine that has a lot of challenges.**

Respondent: You can't run away from this kind of problem in the hospital.

We had a challenge with Ceftriaxone brands. The potency of different brands meant that you had to change doses of each brand, like doubling it if it was to work. We reported these issues to the National Drug Authority, who never gave us feedback. Another was gentamicin was recalled due to several reports of adverse events from using it. We have never gotten supplies again. Remember, if you go to NDS records, you realise that they recalled gentamicin about a year ago.

**Interviewer: How has your MTC solved the issue of stock out? Are you faced with some issues of stock outs? And how have you handled them?**

**Respondent:** Stockouts are sometimes on and off since we are a referral hospital. We handle referrals from lower facilities that put a burden on the limited stock planned for our hospitals. However, stockouts are not as rampant as they used to be since we always factor in referrals in our procurement plan. This has reduced the impact of referrals on our stocks in the hospitals. Patients have to go out of their pockets if we run out of medicine. We may also handle stockout by looking at the medicines in the lower facilities. For example, if we ran short of folic acid, we would try to connect to lower facilities and do what we call redistribution across the district, talk to some of our colleagues in the supply chain and see whether they have this in excess, and if so can you help us for now and we replace later as you wait for your cycle time.

Another way we have handled this is by sending emergency orders. Not often, though. Therefore, there are three ways. The distribution you understand. People were getting out of their pockets and emergency orders. Therefore, we have done so far to minimise the stockout and reduce the expiration of more medicines.

**Interviewer: How has the committee on antimicrobial stewardship practices improved e antibiotic use in the various wards?**

Yeah, it is the one area where we have lagged in the MTC in our hospitals. The gear went down when the head of the committee was transferred, and we had just started some interventions on improving rational antibacterials. We conducted a few CMEs on antibacterial use in the hospital. In addition, we have instituted restrictions on our reserved antibiotics in the second-line and third-line.

It requires a bit of evaluation to see if the strategies are working. We have talked about it in our MTC meetings, but I can only tell you that the only practical thing we have done is just come up with that policy. It is also coming up with a policy for drug promoters because it was also one of the areas that was facing challenges.

**Interviewers: Okay, tell me more about the policy on drug promoters!**

**Respondent:** However, we decided that if you are coming in to promote drugs at the hospital, you have to pass through MTC structures for vetting before being permitted to conduct a CME. Then, the hospital will call morning assembly assemblies of staff. Then we assign your time to come and talk about your products for every staff who could have attended that morning assembly to get some education so that we together get the knowledge because we know that drug promoters also play a role. They give you new abilities that are coming up that you need so that every staff is even beyond one's speciality. When a participatory CME is conducted with all staff in attendance, it allows all members to vet the drug and see whether it's worthy of requesting. Yeah, that is what the MTC has done in that area through an appointed subcommittee, which worked on it. We make small subcommittees to work on that; then, they present their work. Moreover, we approved the policy for the hospital.

**Interviewer: How has the MTC antimicrobial stewardship committee handled the problem of antimicrobial resistance?**

**Respondent:** It is quite challenging to answer because it requires evidence that demands us to carry on culture and sensitivity. We were supposed to start culture and sensitivity because of deficiency in our labs, and we said we would support them with what discs/plates. We sometimes lack that because NMS sometimes does not give us all that and then sometimes they bring it, so it is not regular that we do very serious grounded culture sensitivity for serious antimicrobial stewardship. We somewhat lack the support because, you know, we do not have enough resources, so we only do it on an elementary level.

**Interviewer: Are there activities you are doing to improve the use of antibiotics? Are there some activities you are generally doing in the hospital?**

**Respondent**: Basically, we do what we can manage to ensure we preserve the few antibiotics on our formulary list. We try to emphasise that our doctors use clinical guidelines when treating our patients. Also, we try to restrict some particular antibiotics to the senior consultants; we don't allow them to be used or prescribed by anyone apart from physicians. We try to discourage polypharmacy regarding people's conditions. In summary, discouragement of polypharmacy, the emphasis on the use of guidelines and trying to restrict some classes of medicines to some elect practitioners as much as we would have preferred to do sensitivity tests and see what exactly should be drugs of choice in our setting.

**Interviewer: How confident are you about the MTC tackling the medicine use problem?**

**Respondent:** Our MTC has conducted several activities to improve antibiotics. Even the hospital staff feel that ever since its establishment, the MTC has tackled the issues of medicines availability, thus reducing out-of-stock and medicines wastage. The MTC conducts efficient procurement planning, allowing every department to suggest new requests. They have widened the range of antibiotics in the departments. At the hospital, we now feel that there are reasonable amounts of medicines available at the hospital through the MTC, and the interventions in procuring equipment have been good. The feedback from the hospital staff affirms that the MTC has done something that they feel they associate with and appreciate.

**Interviewer: What has kept you motivated as MTC members?**

**Respondent:** Yeah, we have no lunch or breakfast provisions during the meeting. Most of the motivation has come through the increasing availability of medicines in different wards. There are tangible achievements through increasing the participation of members in suggesting the request for or deletion of the medicine. It has also increased equipment availability, making people comfortable at work.

**Interviewer: How has the MTC enhanced the procurement of health supplies?**

**Respondent:** Yes, we have done a lot as MTC, advocating for the renovation of the records department. We influenced the procurement of the ultrasound, patient monitors, trolleys, and dental chairs. It has come to a place where it will be forwarded to the MTC when there is a need for something to be followed up. Now, all hospital staff appreciate our importance in improving the working environment.

**Interviewer: Do you feel that the MTC now should come to the organogram of the hospital as an important committee?**

**Respondent:** The hospital already acknowledges the MTC as a vital committee, which is displayed in the hospital administration structure. However, what we have been thinking through with the administration is allocating a budget to the MTC for their yearly activities to be run. We are hoping we can grow to this level.

**Interviewer: Are there some challenges you are facing as the MTC, that you can highlight to me as we wind up?**

**Respondent:** financial challenges, especially for the secretary, I have to mobilise and coordinate activities and facilitate and sustain the meetings. Human resource staffing is also a challenge; transfers and retirements disrupt MTC activities, and we lose active members with experience, so we must train a new person. This interrupts the operational rhythms and drags us behind.

**Interviewer: What is the perception of the other staff members at the hospital regarding MTC?**

**Respondents:** At the start, we were regarded lightly, but with the results, they are looking to us as a reference point and with appreciation, as we are currently the most active committee in the hospital, but the challenge is now in sustenance…

**Interviewer: This has been the case with other hospitals, and recently, Uganda recognised all registered MTCS in regional referrals in a booklet. Did you have access to that booklet?**

**Respondent:** I haven't, but it should be in my office as most things were jumbled up in a switch from one station to another. But you can send it to me if you have a soft copy.

Yeah, I am satisfied with what we have achieved so far, but I know there is room for mo. We cannot stay on yesterday's achievements that we fail to see what we can do tomorrow.

Interviewer: Thank you for this interview.

**Pharmacist KI #04**

Age: 37

Sex: Male

Department: Pharmacy

Current position: Senior Pharmacist

Years in practice: 21

Duration of interview: 30:08 minutes

**Interviewer: How long have you had medicines and therapeutics committees (MTC)?**

**Respondent:** MTC has been around since 2012 that is roughly eight years

**Interviewer: What is your role in MTC?**

Respondent: I am the secretary of the MTC

**Interviewer: who are the members that make up your MTC?**

Respondent: The MTC has membership from the pharmacy, and the other major clinical departments are paediatrics, surgery, medicine, obstetrics, and gynaecology, and some are from the administration and the laboratory.

**Interviewer: How does the MTC choose its membership?**

Respondent: Yes! We had to balance the representation of the wards of the Departments with our people's attributes, which was rejected since it would increase the membership.

Initially, the first criteria used was departmental, where we looked at departments like paediatrics; we selected a paediatrician. We wanted an OBS/GYN person. We needed someone from the theatre because usually there are drugs. We needed someone from the nursing section, the head of nursing. We need someone from the stores. We need a pharmacist who oversees his people. We need someone from the administration who is a senior hospital administrator for oversight of administration and just may not actively participate, but they give mmmm…. Ideally brings oversight over so many committees we are there. Then we have an additional official, such as the hospital director herself. We needed a health worker from each department or ward who interfaced with medicines or was very conversant about drugs or medicines management and medicine use. We also looked into the attributes of a person. You look into the person apart from them being a part of the department. Does he promote rational use of medicines and commitment to other tasks? We did not care that the specialist health workers can be nurses, doctors, or anyone who interfaces with medicines and has hands-on experience with medicines. Now we also want healthcare workers who have a love and interest in MTC activities.

**Interviewer: How many members do you have in the MTC? At what level of qualification are they?**

**Respondent:** We currently have 13 members; most of them are medical doctors, and we have nurses and clinical officers. As I told you, we are selecting many members with interests other than high qualification

**Interviewer: How often do you members of the MTC meet?**

**Respondent:** Our members meet once every month to discuss matters arising in their departments

**Interviewer:** What are the major topics of discussion?

Respondent: We usually discuss procurement planning, accountability issues, medicines use problems

**Interviewer: What are the main functions of the MTC?**

Respondent: MTC is very involved in supply chain management and procurement planning of hospital medicines., For the hospital. It has an antimicrobial stewardship subcommittee. It also has a pharmacovigilance committee and subcommittee. It has a drug receiving committee, people who monitor what drugs NMS has received. It has an audit committee.

**Interviewer:** What criteria are used by your hospital when selecting antibacterials?

**Respondent**: We have used pharmacoeconomics as one of the criteria when selecting medicines. In this approach, you have a very expensive antibacterial. It is very efficacious and can produce excellent results, but it will take away 50% of your budget for the patients. What will you do with that drug? You will not bring in such medicine. So, we want to ensure that we get the drug that can serve most patients at a cost that we can afford. But now, we have to strike a balance between cost and benefit. We strike a balance.

**Interviewer**: Okay, would you say the method of selecting medicines has also helped you solve some problems that you usually encounter as medicine use problems?

**Respondent:** Yes, we carefully select as few as possible and have so many of those few antibiotics instead of having a wide range of antibiotics or medicines. We don't have stockouts of antibiotics that is on our formulary list. We go after that, which is on our list.

**Interviewer: How have the criteria of medicine selection helped increase the availability and use of antibiotics?**

Respondent: So, unfortunately, yeah, there is a criterion. We are given a big list from National Medical Stores (NMS). NMS draws this list from Uganda's essential drugs list. So we are given an extensive list, and then they tell you what we can offer you. So you choose to use a template they provide you. So, for example, you look at the options for mental health. Using consumption data, we choose the most "vital or essential" drugs in mental health by deciding from the options given. For example, there can be a debate on the injection of Fluphenazine and Haloperidol. These two drugs serve the same purpose, though they are in different classes. But the costs. One costs four times. That is, Haloperidol is like four-time the cost of Fluphenazine. So you sit down with the department and have these talks and say that to minimise the costs, it is better you use this other drug to minimise the spending on your items. So it starts with a master list from NMS, and then you begin choosing the best option you can work with.

**Interviewer: How has the MTC helped increase the availability and use of antibiotics, particularly antibiotics, since I'm interested in them?**

Respondent: For antibiotics. For example, as an MTC, we shall sit down and use the VEN analysis. For example, let me give you an oral antibiotic. We use Amoxil, Ampiclox, Ciprofloxacin, and Metronidazole for oral antibiotics. Yeah, for children, we have injectables like Ampicillin and Benzylpenicillin, which we also consider. In a scenario of VEN analysis, we consider most injectable, so we first select Ampicillin and Benzylpenicillin then as vital antibiotics for children. Then we choose the essential antibiotics that may include some orals and other necessary medicines like syrup for children.

**Interviewer: Okay. So are there any procedures or guidance you have as the MTC was given on using these antibiotics?**

**Respondent;** Respondent: We stock a few antibiotics that support a large number of people. We wanted to issue medicines to so many people, but we left out the more expensive versions like Cefixime because they would blow up your budget. So, we go for the cheapest variants, antibiotics that can serve most of the population?

**Interviewer: How common is the issue of antibiotic resistance? Is it very common? Do you get to meet it quite often?**

**Respondent:** We have been referred to children who are sickler and have used ceftriaxone for a long time. When we do a culture and sensitivity testing, if confirmed resistant, we recommend the reserved antibiotics, which are our second and third line, like Pisa and Meropenem, for such people. Now, the third and second lines are reserved based on the level of speciality. The children people who are eligible to use them are the people who have also failed on our Ceftriaxone and Metronidazole. Before using the requirement, culture and sensitivity testing must first be conducted. It is also recommended that we do cultural sensitivity before bringing out the guns reserved: Third line Piperacillin and tazobactam or Meropenem.

**Interviewer: Are there some SOPs or guidelines on how someone would prescribe such an antibiotic as a medical officer?**

**Respondent:** Yes, we have an SOP set by the MTC about using these last-line medicines. All the health workers know that these medicines are strictly after culture, and sensitivity shows that they have a call for them, and you need approval from the specialist on the ward.

**Interviewer: So there is this issue of non-formulary use. Are there some antibiotics used out of the formula list, and how have you been guided about them?**

**Respondent:** of course, that happens. It cannot be eliminated. Now, that comes because these are pharmaceutical Representatives who infiltrated our prescribers and told them to prescribe those drugs that are not on our hospital formulary. So we try to talk to these people and know they are hard since they have already been given an incentive. They defended their position and say this is what I felt the patient needed as opposed to what we have. They will measure a patient and know they can afford it. These people do these things knowing the drug is not available in the hospital, but because they have been given incentives from these drug representatives, they prescribe.

**Interviewer: So, on the issue of stewardship, do you have an antimicrobial stewardship committee under your MTC?**

**Respondent** Yes, we have one, but its members have had some training on implementing antimicrobial stewardship interventions. They have gone to every ward enforcing restrictions on the use of third-line antibacterials. You see, the drugs deal with a very narrow range, as in we are dealing with ceftriaxone. Our main antibiotic here is ceftriaxone, which has to be preserved and protected through antimicrobial stewardship. We are not like a Case clinic with all types of antibiotics, and you would want to monitor and account for all the antibacterials we stock. But here you have one option. Everyone is taking ceftriaxone. Someone comes in with a cough, and even someone comes in with Covid. We start with ceftriaxone.

**Interviewer: so there is an issue of overuse of such a drug,**

Respondent: Yes, it is like a song. So, we'll start a youngster on antibiotics and then send for blood cultures since we don't have any other alternatives. For example, they may advise starting ceftriaxone simultaneously as CBC, blah blah. Even someone with Malaria may begin taking ceftriaxone while the tests are being performed.

I**nterviewer: So is there any intervention you are thinking about reducing the misuse of ceftriaxone?**

**Respondent**:

However in my opinion. There is also 5% to 10% of medicines wasted on through empirical treatment. Empirical treatments that, most times, even this empirical treatment. You see, they do the empirical treatment because they start this empirical treatment even before the blood culture comes back. The turnaround time for some of these tests is too long, possibly four days. You are not going to keep someone on the hospital bed for four days before you start them on anything, you understand? So sometimes, this practice has stuck because it has given us benefits and does work. It is not like……

**Interviewer: How has the policy of one single drug in large quantities, and it is availed to many patients, saved you from stockouts**

**Respondent:** The policy of stocking a few drugs in large quantities has saved us from stockouts. The one thing is for sure, we have never had a stock out of ceftriaxone, never! It is cheap, and it is largely available.

**Interviewer: How has the MTC reduced the spread of antimicrobial resistance?**

Respondent: Once you abuse antibiotics, for example, during empirical treatments, you cause pressure on the antibiotic. For example, giving ceftriaxone to someone who doesn't need it increases the development of resistance. If this practice continues without control in the coming years, we may have no antibiotics. In this hospital, our laboratory services have let us down. They are not up to speed. One General laboratory is getting samples from all corners of the hospital wards. And even from the district hospitals, we get samples from outside. So the turnaround time is not that good.

**Interviewer: What capabilities does the MTC have in dealing with antimicrobial resistance?**

Respondents: We have been trained on these aspects of implementing antimicrobial stewardship intervention to combat antimicrobial resistance, but workload has been a limitation since these MTC roles are assigned to us outside our core duties. So you find your core role is taking up 90% of your day. So this other MTC role is left time, unless you are truly passionate about it, you find yourself not vigorous about it. For example, antimicrobial stewardship. I think it's a lost war, and there are some things you would want to start to win. Do you understand?

**Interviewer: What is your comment on the government owning the National Committee, antimicrobial stewardship committee, and representation, from all your hospitals? Do you think it would change the negative aspects we are facing?**

Respondent: Yeah, of course! If the government owns it. That is the other thing that I always tell these partners. You come here, you come to Kabaale, and you are doing antimicrobial stewardship for us. Then you go away with the data and present it at National workshops and forums. First, we are going to look at how you collect your data. We don't even know the meaning of the data of collect. So there is no ownership of the whole activity. So, if the government takes it up from the top and claims ownership of stewardship, it's a step in the right direction.

**Interviewer: Did you follow any guidelines while you were establishing your MTC?**

Respondent: We followed the WHO manual for establishing MTCs. Though we are told the Ministry of Health has established an MTC manual in the Ugandan context, we have no copy yet. Right now, we are required to send an annual report to the Ministry on the performance of MTC, indicating that the government is taking ownership. A study like this one will go a long way in disseminating our work.

**Interviewer: What kind of training and support is needed for MTC?**

Respondent: We have people with skills in logistics management, antimicrobial stewardship intervention implementation, and adminstration. We also want MTC to be supported like other hospital committees. Like, when it comes to this hospital, everyone knows that the contracts committee is where the money is. Okay? In the top management committee, there is some money. There are some committees where the administration feels that these people are important so the administration gives them some money. But MTC is one of those dead committees whereby there is no money! It is a dry one.

**Interviewer: What kind of motivation keeps members attending these meetings besides money or snacks?**

Respondent: As I have told you, most members attend MTC meetings and go for four hours without even a bottle of water. Members are learning that the meetings are beyond a snack or a soda since their department issues can be attended to due to their attendance. When we sit or call meetings for members to suggest which drugs to order for, most endeavour to attend and those who do not come, no one fights for their department. So from experience, No one is going to fight for your rights as a department. For example, the dental department here does not usually come, and we can spend six months without ordering dental items. And when they complain, you ask them if you came to the meeting. We called you to tell us your needs for the meeting, but you did not come. And then they shut up! So from that experience, sometimes people come for fear of, like, if I do not come…

**Interviewer: How have you harmonised all the needs in another department?**

**Respondent:** This is an important question. To avoid pulling ropes with several departments, where everyone starts demanding that I need more of this, put more money into this, put more money into that. For drugs, we depend a lot on enabling departments to determine what medicines they need in severe conditions and what they need in day-to-day conditions. This is like evaluating medicines in the VEN system, where all departments must agree upon the "vital and essential" and serve a wide patient population. We stock these in large quantities to avoid stockouts. Consensus is easy in selecting such drugs because I have enough membership support.

Most members were given appointment letters, and as MTC, we have onboard all the staff. We want to make decisions that are agreeable to other members of departments. They give us a pat on the back, soda, and motivation when you meet them.

**Interviewer: What functions will the MTC focus on in the future?**

Respondent: Well, shortly, we had planned to go and Benchmark with other MTCs. We wrote a letter to the administration to fund us to go to Lacor because we heard that Lacor Hospital has a very good MTC. But like I told you, the hospital administration did not view this as very important. So they did not fund the expedition.

**Interviewer: What factors in your environment would you think could help the MTC do its work better?**

**Respondent:** The administration granted the secretariat of the MTC an office. This has increased publicity of our work in the hospital. Most staff come and consult on the procedures we have developed and guidance as well.

**Interview: Has the administration included the MTC on the hospital organogram?**

Respondent: yes, but The organogram has posts of people, not committees. There are no committees on the organogram. Not at all; there are individuals.

**Interview: In your view, Do you think the MTC activities have changed the perception of health workers on medicine use?**

**Respondent**: Okay, people did not understand why they didn't get some medicines in the past. But ever since activating the MTC people, no longer…. The complaints have gone down because they know that…. for example, recently, someone was asking for Tranexamic acid. And I told him point-blank. It was an OBS/GYN person. I told him your department was represented in the MTC meeting when we were doing procurement planning, and your need for Tranexamic acid did not come up for., so do not expect it to come for this whole year, and that was solved as simple as that.

Yeah, this is important because no one is going to come out of the blue and start asking for a rare drug when they know they didn't plan for it. Yeah, that's very critical. For example, these theatre people recently complained about some theatre drugs that you're not getting. And when I told them who represented them, they were very bitter about the person who represented them in the meeting. They were like that one does not work. Why did you come to that meeting? I told him. Okay, for the next meeting, you should send someone who works.

**Interviewer: Are you satisfied with the work done by this MTC so far?**

**Respondent:** So far, I am 50% satisfied. We could do much more if we were empowered to do much more. But for the little inputs that it has, it's okay. It is better than being nonexistent,

Interviewer: Thanks for the interviewer. It was an engaging interview we've, and as a requirement from my degrees, ethics and research committee, I have to compensate this time with the twenty thousand Shillings.

**Pharmacist KI #05**

Age: 38

Sex: Male

Department: Pharmacy

Current position: Senior Pharmacist

Years in practice: 15years

Duration of interview: 30 minutes Gender: Male

**Interviewer: How long has MTC been in operation?**

**Respondent:** We have been in operation since 2011.

**Interviewer: What is your role in the MTC?**

**Respondent:** I am the secretary of the MTC, where I have a role in planning for the meetings and taking minutes. Do a follow-up on the resolutions of the committee.

**Interviewer:** What are the major functions of MTC in Mubende Hospital?

**Patrick:** Okay, uhmm, We conduct a lot of supply chain management to ensure that medicines are available to the hospital. We also develop guidelines for adding or deleting medicines in the supply chain management. We have pharmacovigilance to adverse drug reactions and medication errors such as administering wrong doses, indications or duration. We have also started antimicrobial stewardship though it is a rudimentary stage.

**Interviewer: How does the MTC choose its membership?**

**Respondent:** The medical director appointed specialists from medicine, paediatrics, surgery and obstetric and gynaecologist, pharmacist, and nurses. The chairperson is a consultant surgeon, Then there is also a physician from the medical ward. Then there is the head of nursing, an administrator, and then the head of medical records.

**Interviewer: What criteria do you use for membership of MTC?**

**Respondent:** We choose our membership from hospital departments, administration and head nurses. For one to be a member, he has to be available and then knowledgeable of the department's needs. He must have been in the hospital for more than two years.

**Interviewer: What are the criteria used by MTC in selecting medicines?**

**Respondent:** We look at the prevalence of the disease. Then, we also considered a special category of patients like pregnant mothers and Children. We also look at common comorbidities.

**Interviewer: Is there some special consideration you also give, or it's the same criteria for antibiotics?**

**Respondent:** The selection of antibiotics on the formulary is guided by consumption, and the national essential drug list also guides us. Of course, heads of departments are also at liberty to suggest antibiotics they request for addition, and they are permitted to prescribe medicines which are outside the essential drug lists.

**Interviewer: How have your selection criteria helped in availing yourself of antibiotics all the time in the hospital?**

**Respondent:** We use the VEN system to ensure we have vital, essential antibiotics. We stock these antibiotics in large quantities. We follow our procurement plan as the committee but normally consult the National Medical Stores for guidance.

**Interviewer: Okay. So, are there some procedures to put in place as MTC to guide or improve the use of medicines in the entire hospital?**

Respondent: Normally, contracts are signed by the consultant. These other ones can also be prescribed, but the consultant has to countersign to request from the pharmacy. The adopted UCG (Uganda clinical guidelines ) will be used in specific conditions that need treatment using specific antibiotics. Hospitals are developed guided by the

**Interviewers: Okay. How do you handle the issue of non-formulary use of medicines within the hospital non-formulary?**

Respondents: We usually advise the clinicians on the alternatives available.

**Interviewer: What has been the influence of drug promotions on the way medicines are used in your hospital?**

**Respondents**: Yeah, they have an influence, whereby you find some clinicians using brand names to prescribe, so, hmm, in that case, we have always tried to encourage the physicians to use the generic, although compliance is not 100%some will still prescribe the other branded name.

**Interviewer: Have you experienced antibiotic resistance in the hospital? Have you come across issues of antibiotic resistance as MTC?**

**Respondent**: Especially in the current one, we have registered resistance to cephalosporin, especially ceftriaxone. Because we can do culture and sensitivity, IDI is supporting us, and they give us the reagents.

**Interviewer: Have your members received training on antimicrobial stewardship?**

**Respondent:** Not yet, but we had a meeting last week. It was last week. Unlike members from MTC, one of the doctors from IDI will train the different categories of health workers in the hospital.

**Interviewer: How has the MTC antimicrobial stewardship helped in medicine use, that is, the interventions that can improve the quality of patient care?**

**Respondent:** hmm. We intend to do that during the... Okay, we started with the especially the adverse drug reactions, improving the knowledge of the staff on ADRs, but that one was limited to patients who are …okay, patients who are on ARVs and anti-TBs.Patrick: The staff working in those units have received two training pieces so far.

**Interviewer: Are members of your MTC competent to conduct committee activities?**

Patrick: I'm confident they can execute all the duties. We also have the... On the other side, I didn't mention the lab technologists. They are actively supported in these activities.

**Interviewer: What kind of activities do you look to perform MTC in the future?**

Respondent: yeah, we want to be doing medicine use reviews, where Maybe you can pick a particular category of patients, say those who are on maybe oral anti-diabetic and review to see whether there is a response to the treatment

**Interviewer: What do you think would be happening without the MTC?**

Patrick: We would be having much irrational use of medicines and rampant stockouts. Because there will be no proper selection of medicines

**Interviewer: What is motivating your membership in conducting all these activities they're doing? What is driving them?**

Respondent: They are being given formal appointment letters. so that one motivated them because you can use that to make your CV.

**Interviewer: So what else motivates them?**

Respondent : Patrick: incentives are not there yet.Though they promised that members will be given some benefits,

**Interview: What kind of support may the MTC need?**

**Respondent:** Yeah, the committee would need funding which we are trying to engage the hospital management if a budget can be allocated for MTC activities

**Interviewer: Are you contacting also partners? Some partners for help? Are you contacting also partners?**

Respondent; They have been helping us, but they said they have some financial constraints this financial year. Also, like benchmarking to see what other facilities have also done

**Interviewer: How do all health workers in the hospital perceive your MTC, how is it striking their minds, do they feel it is very important, or it is limiting them?**

Respondent: In the beginning, the attitude was negative. Others could think they are being undermined like they are being considered to be not knowledgeable enough because MTC would go and talk to the staff. And tell them about the need to adapt to the modern practices

**Interviewer:** I would like to thank you so much

1. [↑](#endnote-ref-1)
